# Comparative Analysis of Abrasive Materials and Polishing System on the Surface Roughness of Heat-Polymerized Acrylic Resins

**DOI:** 10.1055/s-0041-1736293

**Published:** 2021-12-10

**Authors:** Stanley Chibuzor Onwubu, Phumlane Selby Mdluli

**Affiliations:** 1 Department of Chemistry, Durban University of Technology, Durban, South Africa

**Keywords:** abrasive materials, eggshell powder, polishing system, pumice, surface roughness

## Abstract

**Objective** The aim of this *in vitro* experiment was to see how the operator's manual skills, polishing equipment, and abrasive materials affected the surface roughness of denture base resins.

**Materials and Methods** Forty polymethyl methacrylate (PMMA) specimens were created and polished by using two different polishing systems, namely hand and automatic polishing machines. Three operators hand-polished 30 of specimens with eggshell powder and pumice, while 10 were automatically polished ( *n* = 5). A profilometer was used to determine the average surface roughness (Ra) after polishing. The Ra values for the specimens hand-polished were analyzed by using paired sample testing. The Ra values for all polished specimens were analyzed by using a one-way ANOVA. Differences between the two abrasive materials as well as the polishing system were determined by using the Bonferonni tests ( *p* = 0.05).

**Results and Conclusion** For the PMMA specimens hand-polished, there was a strong connection in the Ra values. There were also significant variations in the Ra values across the three operators ( *p* < 0.001). The automated technique created a substantially smoother surface than the traditional technique ( *p* = 0.001). The greatest Ra values (0.20 µm) were found in specimens polished traditionally by using pumice, whereas the lowest Ra values (0.04 µm) were found in specimens polished mechanically with eggshell powder. The automated polishing system was the most effective polishing method when the Ra values were connected to the level of smoothness.

## Introduction

For many years, acrylic resin made of polymethyl methacrylate (PMMA) has been the most popular denture base material due to its unique properties such as ease of processing, lightweight, cheap cost, aesthetic qualities, and stability in an oral environment. 1 2 Despite this, the surface properties of PMMA denture base material are poor, 3 which could subsequently act as a substrate for microorganism adherence and biofilm formation. 2 Since PMMA denture base materials are used in the oral cavity, a smooth and highly polished surface on an acrylic resin denture base is essential for maintaining dental health and preventing bacterial colonization. 4 5 However, it is not apparent how best to get such a surface.

Tupinamba et al 5 believe that correct polishing is essential in preventing bacterial retention and plaque build. Other investigations 6 7 8 have found that the clinical quality and success of dental prostheses intraorally are determined by a well-polished and smooth denture surface. In addition, in vivo investigations 9 10 11 have demonstrated that after polishing, the surface roughness of PMMA prosthesis should not exceed 0.2 µm.

PMMA dental prostheses hand-polished traditionally with pumice on a laboratory lathe machine create a surface roughness that surpasses the threshold value of 0.2 µm according to some authors. 12 13 Onwubu et al 14 recently showed that eggshell powder with particle sizes ranging from 15 to 0.3 µm may be employed as a substitute for pumice in reducing the surface roughness of PMMA base resin below the 0.2 µm of threshold limit value. However, operator variability, according to Abuzar et al, 15 can affect surface roughness values (Ra), which can lead to higher values in clinical practice. The goal of this in vitro experiment was to see how the operator's manual skills, polishing equipment, and abrasive materials affect the surface roughness of dental prostheses that were traditionally polished with eggshell powder and pumice using a laboratory lathe machine in comparison with those polished automatically.

The following hypotheses were tested: (1) there is variation in operator manual skills in decreasing the surface roughness of PMMA base resins; and (2) there is a significant difference in the abrasive materials employed and the surface roughness values measured (3) surface roughness values produced by the automatic polishing system significantly differs from the traditional laboratory lathe machine system.

## Materials and Methods

Pumice (Navajo) was acquired from a local outlet in South Africa, and eggshell powder was created by using the process indicated by Onwubu et al. 16 17 18 Sodium lauryl surfactant (0.5 g) was added to 20 g of blended eggshell powder to improve its wettability. The mixture was further milled following the procedure reported by Onwubu et al. 16 17 18

### Scanning Electron Microscope

A scanning electron microscope (S-3000N-Carl Zeiss) operating at controlled atmospheric conditions at 20 kV was used to characterize the surface morphology of the eggshell powder and pumice. Before SEM observation, small quantities of the powders were spread in the sample holder and then coat sputtered for 30 minutes to prevent a build-up of electrostatic charge.

### Preparation of Polymethyl Methacrylate Specimens

A total of 40 (15 × 15 × 3 mm) heat-polymerized acrylic resin specimens were created. All specimens were polymerized as per the manufacturer's instructions (Vertex-Dental BV). At 18,000 rpm, a tungsten carbide bur (Cross-cut, coarse – ISO no. 500104237065; Bredent GmbH & Co KG) was used to trim the specimens. All specimens were completed with abrasive paper before polishing (CC768 Silicon Carbide; Deer Abrasives).

### Automatic and Conventional Polishing of the Polymethyl Methacrylate Specimens

Three operators (skilled dental technicians) hand polished 30 specimens using a laboratory lathe machine at 1,500 rpm for 2 minutes each ( *n* = 5), with eggshells and pumice as the two abrasive materials ( Table 1 ). The water and powder consistency was achieved by mixing 30 g of powder with 5 mL of water to form a slurry. On the other hand, 10 specimens were automatically polished ( *n* = 5). The specimens were inserted in a mounting resin prior to automatic polishing (AMT composite). A silicone rubber mold was used as the mounting foundation for the specimens during the embedding preparation. PMMA specimens were then inserted into the mold. Part A and Part B (Composite) were mixed in a disposable plastic cup to make a fast-setting resin, which was then poured into the mold in a 1:1 ratio. The embedded resin was removed from the silicone mold after 2 minutes. The procedure and process of automatic polishing are detailed in Onwubu et al. 4

**Table 1:** Polishing process and sample size

Operator(s)	Polishing materials	Polishing system	Number of specimens	Revolution per minute	Time (min)
Operator 1	Eggshells and pumice	Laboratory lathe (RENO)	10	1,500	2
Operator 2			10	1,500	2
Operator 3			10	1,500	2
Automatic machine		RotoPol-35, PdM-Force-20; Struers	10	300	2

### Surface Roughness Analysis

Using a Wintrace surface analysis system profilometer, the surface roughness (Ra values) of PMMA specimens was measured (Taylor Hobson Ltd). A 0.8-mm cut-off filter, a 4.00-mm evaluation length, and a range of 5.1 µm were used to calibrate the profilometer. Each specimen had five measures of surface roughness, and the statistical analysis was based on the mean average Ra values.

### Statistical Analysis

The Shapiro-Wilk test was done to evaluate the normality of the Ra values by using software (SPSS v27; IBM Corp). The paired sample test was used to examine the intragroup mean differences of the polished PMMA specimens polished manually by various operators, while the independent sample test was utilized to evaluate the intergroup mean differences of the polished PMMA specimens. To compare the polished surfaces of the PMMA specimens in the automated polished group, a one-way analysis of variance (ANOVA) was employed, which followed by the Bonferroni test ( *α* = 0.05).

## Results

### Surface Morphology of Eggshell Powder and Pumice

**Fig. 1:**
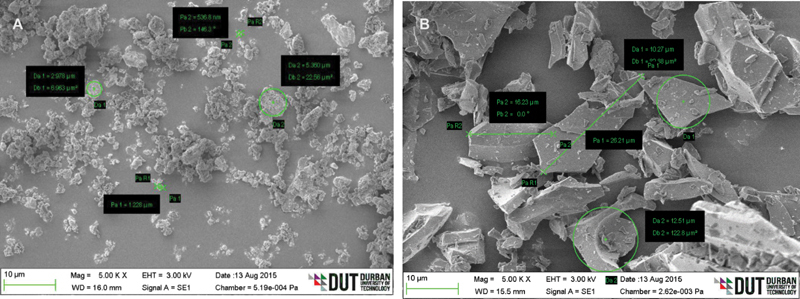
Scanning electron microscope images showing ( **A** ) eggshell powder, and ( **B** ) pumice showing particle.

The eggshell powder particles have an irregular form and a sponge-like structure, as seen by the SEM image ( Fig. 1A ). The image reveals that the eggshell powder had uneven particle size distribution in a range of 1.9 to 568 nm. O'Brien 19 believes that this uneven particle form is beneficial in creating better-polished surfaces. On the contrary, the SEM image for pumice reveals triangular like particles ( Fig. 1B ). The particle distribution for pumice reveals an uneven distribution in a range of 10 to 26 µm.

### Operator's Manual Skills Analysis

The normal distributions of the operator's manual skills on the R _a_ values of the polished specimens are presented in Table 2 . The Shapiro–Wilk test for normality showed no significant differences against the normality of the R _a_ values ( *p* > 0.05).

**Table 2:** Normality test

Operators	Type of abrasive material	Shapiro–Wilk test		
		*df*	*p* -Value	Level of significance
Operator 1	Eggshell powder	5	0.754	Not significant
	Pumice	5	0.141	Not significant
Operator 2	Eggshell powder	5	0.135	Not significant
	Pumice	5	0.272	Not significant
Operator 3	Eggshell powder	5	0.814	Not significant
	Pumice	5	0.215	Not significant

The paired sample test for the intra-mean comparison results is illustrated in Table 3 . A positive strong correlation was found in the surface roughness for the PMMA specimens polished manually by the different operators. An examination of the R _a_ values means for operator 1 and operator 2 for example (combined pumice and eggshell powder) indicates that the R _a_ values of operator 2 (0.10 ± 0.06 µm) were significantly higher than the operator 1 (0.15 ± 0.06 µm). Similar pattern is observed for operator 3 (0.13 ± 0.07 µm) and operator 1 (0.15 ± 0.06 µm), as well as operator 2 (0.10 ± 0.06 µm) and operator 3 (0.13 ± 0.07 µm).

**Table 3:** Intragroup mean comparison

Operators		Mean	*n*	± SD	± SE	Correlation	*p* -Value
Pair 1	Operator 1	0.15	10	0.06	0.02	0.866	0.001
	Operator 2	0.10	10	0.06	0.02		
Pair 2	Operator 1	0.15	10	0.06	0.02	0.926	0.000
	Operator 3	0.13	10	0.07	0.02		
Pair 3	Operator 2	0.10	10	0.06	0.02	0.831	0.003
	Operator 3	0.13	10	0.07	0.02		

Abbreviations: SD, standard deviation; SE, standard error.

The intergroup mean comparison of PMMA specimens polished with different polishing materials by various operators is shown in Table 4 . Overall, the Ra values of the eggshell powder-polished specimens were substantially lower than those of the pumice-polished specimens ( *p* < 0.001).

**Table 4:** Intergroup mean comparison

Type of Abrasive material		*n*	Mean	± SD	± SE	*p* -Value	Significance
Operator 1	Eggshell powder	5	0.0940	0.02074	0.00927	0.000	Significance
	Pumice	5	0.2020	0.02950	0.01319		
Operator 2	Eggshell powder	5	0.0520	0.01095	0.00490	0.000	Significance
	Pumice	5	0.1520	0.02168	0.00970		
Operator 3	Eggshell powder	5	0.0660	0.01140	0.00510	0.001	Significance
	Pumice	5	0.1880	0.03493	0.01562		

Abbreviations: SD, standard deviation; SE, standard error.

**Fig. 2:**
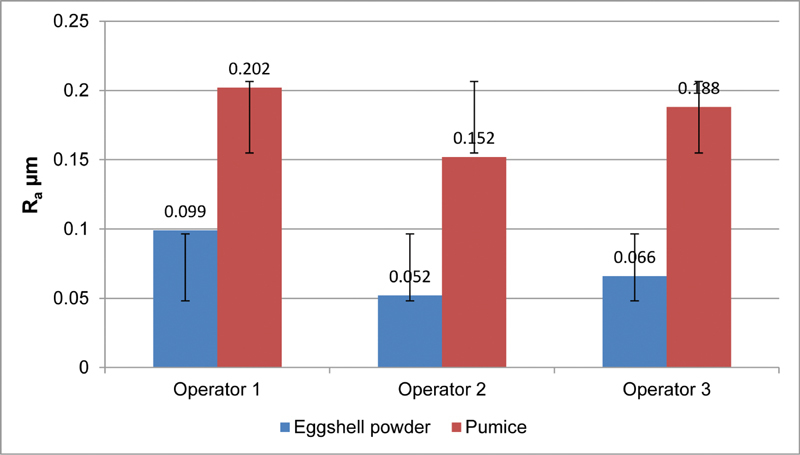
This shows the variations in the mean value of surface roughness received from the operator using various polishing products.

After 2 minutes of polishing, differences in mean surface roughness (Ra) of PMMA specimens hand-polished with eggshell powder and pumice abrasive materials by 3 separate operators (n =5; Fig. 2 ). PMMA stands for polymethylmethacrylate (methyl methacrylate).

### Automatic Versus Conventional Polishing Analysis

Table 5 illustrates the R _a_ values obtained from the profilometry analysis of the PMMA specimens that were automatically and manually polished. As revealed by the R _a_ values, uniformity and consistency in the polishing were observed for the specimens that were automatically polished with pumice and eggshell powder. In contrast, inconsistencies were observed in the R _a_ values, particularly in respect to the PMMA specimens conventionally polished with pumice.

**Table 5:** The surface roughness value of polymethyl methacrylate specimens polished with eggshell powder

	S/N	Conventional polishing system (R _a_ µm)			Automatic polishing system (R _a_ µm)
		Operator 1	Operator 2	Operator 3	
Eggshell powder	1	0.12	0.07	0.06	0.03
	2	0.08	0.05	0.05	0.05
	3	0.09	0.05	0.08	0.04
	4	0.11	0.04	0.07	0.03
	5	0.07	0.05	0.07	0.04
Pumice	1	0.25	0.13	0.22	0.06
	2	0.19	0.14	0.21	0.06
	3	0.18	0.17	0.13	0.06
	4	0.21	0.18	0.19	0.05
	5	0.18	0.14	0.19	0.06

Table 6 shows the 1-way ANOVA, mean, standard deviation, and standard error data. Surface roughness for PMMA specimens and their interactions with abrasive materials, as well as the polishing method employed in the polishing process, were found to be significantly different ( *p* < 0.001).

**Table 6:** The ANOVA test for abrasive material and polishing system

		*n*	Mean	± SD	95% CI for mean		ANOVA test	Bonferroni test
Polishing material and system					Lower bound	Upper bound	*p* -Value	
Polished with eggshell powder	Operator a 1	5	0.09	0.02	0.0683	0.1197	0.000	0.000 a d
	Operator b 2	5	0.05	0.01	0.0384	0.0656		0.000 a d
	Operator c 3	5	0.07	0.01	0.0518	0.0802		0.000 a d
	Automatic d	5	0.04	0.00	0.0276	0.0484		
Polished with pumice	Operator a 1	5	0.20	0.03	0.1654	0.2386	0.000	0.000 a d
	Operator b 2	5	0.15	0.02	0.1251	0.1789		0.753 b d
	Operator c 3	5	0.19	0.03	0.1446	0.2314		0.031 c d
	Automatic d	5	0.06	0.00	0.0524	0.0636		

Abbreviations: ANOVA, analysis of variance; CI, confidence interval; SD, standard deviation; SE, standard error.

a Operator 1

b Operator 2

c Operator 3

d Automatic

When it came to the polishing system, the group that was automatically polished with pumice had the lowest Ra mean (0.06 ± 0.00 µm) compared to the PMMA specimens that were hand-polished. Similarly, the group that polished with eggshell powder automatically had the lowest Ra mean value (0.04 ± 0.00 µm). The Ra values of the group polished with pumice were substantially lower than the Ra values of the specimens hand-polished conventionally by operators 1, 2, and 3 ( *p* < 0.001).

The Ra values of the specimens hand-polished traditionally by operators 1 and 3 were substantially lower than those of the group polished automatically using eggshell powder ( *p* < 0.001). The Ra values of the group polished automatically with eggshell powder and operator 2 did not differ significantly ( *p* > 0.05). Overall, the group that operator 1 hand-polished with pumice had the greatest Ra mean value (0.2 ± 0.03 µm), whereas the group that was automatically polished with eggshell powder had the lowest Ra mean value (0.04 ± 0.01 µm).

## Discussion

Previous studies reveal the surface roughness of PMMA resins is influenced by the material's intrinsic properties, the polishing method, and the operator's physical abilities. 20 21 Tupinamba et al 5 note that polishing is a surface treatment that involves the use of appropriate materials and processes. Dental prostheses are traditionally hand-polished in the dental laboratory by using a lathe machine and pumice as the abrasive material. 22 Despite this, Corsalini et al 20 argued for the adoption of an automated polishing system instead of the traditional polishing approach. The influence of operator manual skills, polishing method, and abrasive materials on the surface roughness of dental prostheses was compared in this study. The surface roughness of the PMMA resins was measured by using profilometry with a contacting stylus. The roughness parameter (Ra) was measured, and statistical analysis was performed. The Ra value which corresponds to the average peak and valley distance is an important parameter commonly used to quantitatively describe the surface roughness in vitro. 14 23 Although the Ra does not measure the amplitude and spacing of superficial irregularities, 23 nevertheless, the surface roughness of a PMMA denture base resin is clinically benchmarked using the Ra values. 24

Moreover, and in light of the several clinical studies on surface roughness reported in the literature, 9 10 the results obtained in this study were explained by using the Ra threshold value of 0.2 µm. The first hypothesis was accepted based on the study results. The intragroup data ( Table 2 ) demonstrated that there was significant variability in the operators and the surface roughness (R _a_ ) value of the polished PMMA specimens ( *p* < 0.01). The differences in the surface roughness value obtained from the three operators could be attributed to the inconsistency in hand polishing a denture. 15 20 Corsalini et al 20 noted that the operator's hand abilities are influenced by human variables such as attentiveness and writs trembling, which can affect the surface finish quality. The aforementioned factors could have had an impact on the measured differences in surface roughness.

Overall, the Ra values recorded in all of the PMMA groups hand-polished with eggshell powder were considerably lower than those observed in the pumice-polished specimens ( *p* < 0.001), which led to the acceptance of the second hypothesis. From a clinical perspective, and consistent with Onwubu et al, 14 the lower R _a_ values measured with eggshell powder suggest that the abrasive material is more likely to produce a more highly polished dental prosthesis. More so, the different R _a_ values between the eggshell powder and pumice may be related to the differences in the particle sizes between the two abrasive materials ( Fig. 1 ), which reveal that eggshell power had smaller particle sizes when compared to pumice. It is reported that abrasive materials of smaller particle sizes created newly formed and sharper particles faster during the abrasion process, which in turn reduces the surface roughness of dental prostheses. 25 Also, the inclusion of surfactant in eggshell powder could have contributed to the differences in the R _a_ values between eggshell powder and pumice. Surface-active compounds such as surfactants have been reported to improve the wettability and to enhance the mechanical properties of materials. 26 27

Moreover, the findings of this study demonstrated that an automatic polishing method is more likely than manual polishing to provide a better polished ( Table 6 ). The PMMA group with the highest mean average was hand-polished with pumice by operator 1 (0.20 ± 0.03 µm), whereas the lowest was automatically polished with eggshell powder (0.04 ± 0.01 µm). This could, however, be attributed to the standardization of the polishing process, which minimizes the human factors consistent with hand polishing. 4 20 As a consequence, the third hypothesis was accepted, as the polishing systems used in this study were significantly different in respect to the surface roughness values ( *p* < 0.01).

Furthermore, the findings of this investigation, particularly the hand-polished PMMA specimen, are consistent with those of Srividya et al 13 and Onwubu et al. 14 They reported a mean Ra value of 0.36 to 0.13 µm, which matches the findings of this study. However, Kuhar and Funduk 28 reported a Ra value of 0.79 µm. It is worth noting that Al-Kheraif, 29 who employed a pumice-automated polishing method, obtained results that are comparable to the average Ra values obtained in this study's pumice-automated polishing group. Furthermore, Onwubu et al 14 observed a mean Ra value of 0.07 µm on PMMA specimens hand-polished with eggshell powder (fine = 0.5 µm), which matches the findings of our investigation.

Although the automatic polishing system produced useful and reliable findings in our investigation, it has certain drawbacks. Appliances are created to fit the mouths of individual patients at a dental laboratory. As a result, an automated polishing system cannot be used since denture polishing is never done on entirely level surfaces. To establish the efficiency of the automated polishing system on the surface roughness of dentures made for dental patients, more clinical investigations are needed.

## Conclusion

Within the limitations of the present in vitro study, it can be concluded that the most efficient polishing technology for minimizing the surface roughness of PMMA base resins is automatic polishing. The eggshell powder may be utilized as an alternate abrasive material in hand and automatic polishing of denture base materials. From a dental laboratory perspective, this study is highly useful to dental technicians in the selection of abrasive materials as well as the choice of the polishing system that could produce clinically acceptable PMMA dentures.
